# Extreme Weight Loss and Psychosis as Presenting Signs of Thyrotoxicosis

**DOI:** 10.7759/cureus.14045

**Published:** 2021-03-22

**Authors:** Thu Nhi Le, Ismail Ganim, Eric Landa, Hemal Patel, Talhah Siraj

**Affiliations:** 1 Internal Medicine, Arkansas College of Osteopathic Medicine, Fort Smith, USA; 2 Internal Medicine, Unity Health, Searcy, USA

**Keywords:** thyrotoxicosis, thyroid storm, endocrinology, hyperthyroidism

## Abstract

Thyroid storm is an acute, life-threatening syndrome due to an exacerbation of thyrotoxicosis, which is when you have an excess of thyroid hormone in the body. Thyroid storm can be precipitated by infections, surgery, or untreated thyrotoxicosis. Multisystem involvement is often seen. Typical symptoms include fever and tachycardia, which are rather common, as well as more severe symptoms such as atrial fibrillation, congestive heart failure, hepatic failure, delirium and coma. The Burch-Wartofsky Point Scale is often used for the clinical diagnosis of thyroid storm. Prompt diagnosis and therapy are required to prevent complications and mortality in patients with thyroid storm. Here we present a case of thyroid storm in a patient that presented with psychosis and significant weight loss.

## Introduction

Thyroid storm is an acute, life-threatening syndrome due to an exacerbation of thyrotoxicosis. The most common etiologies of hyperthyroidism include Graves' disease, toxic adenoma, thyroiditis, and multinodular goiter. This case report describes a 56-year-old female with untreated hyperthyroidism who presented for thyroid storm.

## Case presentation

A 56-year-old female with a past medical history of essential hypertension, major depressive disorder, a history of bleeding gastric ulcer, frequent urinary tract infections and morbid obesity with a BMI of 51 arrived at the emergency room due to altered mental status and hallucinations. History was obtained from the patient’s family members. The family reported that the patient was admitted to another hospital two days prior due to nausea, vomiting, decreased appetite, and severe weakness. The patient's mental status was initially normal at that time but after being discharged from the hospital, she developed symptoms of altered mental status with hallucinations. The patient's family then presented to our hospital’s emergency room. On review of systems, the patient’s family reported that the patient has a history of severe depression, 120-pound weight loss within the past year with complaints of hair loss, dry mouth, fevers, poor oral intake, vertigo, and tinnitus. Past surgical history includes a laparotomy band and abdominoplasty. Vital signs showed a blood pressure of 125/65 mmHg, pulse rate of 125 beats per minute, respiratory rate of 16-20 breaths per minute, and temperature of 98°F. Medication review did not yield evidence of agents known to cause thyroid abnormalities. The patient had altered mentation and was tremulous. Her neck was supple with no jugular vein distention (JVD), lymphadenopathy, or carotid bruit. Her mucous membranes were dry. The patient was tachycardic with no wheezes, crackles, or rhonchi. Skin exam showed no signs of jaundice. Extremities showed no clubbing, cyanosis, or edema. Electrocardiogram (EKG) demonstrated Sinus tachycardia. The patient’s laboratory values are shown in Table 1.

**Table 1:** General laboratory data CO_2_: carbon dioxide; BUN: blood urea nitrogen

VARIABLE	VALUE
White blood cells / 𝜇L	4.5
Red blood cells / 𝜇L	3.18
Hemoglobin (g/dL)	8.5
Hematocrit (%)	27.3
Platelets/L	187
Sodium (mmol/l)	139
Potassium (mmol/l)	3.4 (Low)
Chloride (mmol/l)	103
CO_2_ (mmol/l)	18 (Low)
BUN (mg/dl)	11
Creatinine (mg/dl)	0.8

**Figure 1:**
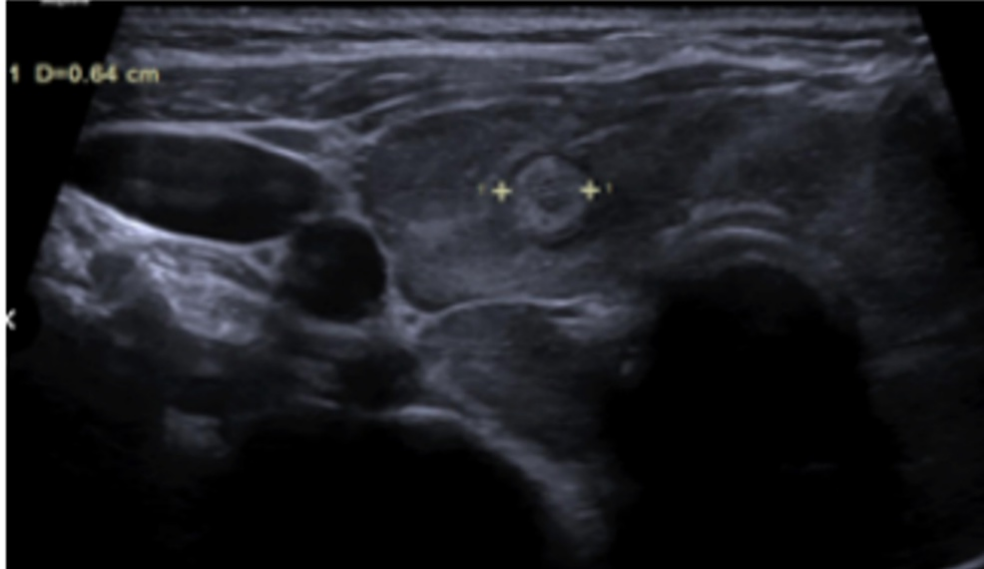
Ultrasound of thyroid. Heterogeneous thyroid echogenicity with increased parenchymal vascularity.

The patient’s urinalysis was negative for a urinary tract infection. Urinary drug screen (UDS) was negative for illicit substances except for opioids. Head CT without contrast showed no mass or hemorrhage. Ultrasound of the thyroid showed two right thyroid nodules, the most suspicious nodule being a solid, hyperechoic, well-circumcised, taller greater than wider, and measuring 0.7 x 0.7 x 0.6 cm. American College of Radiography Thyroid Imaging Reporting and Data System (ACR TI-RADS) score of TR3. There was heterogeneous thyroid echogenicity with increased parenchymal vascularity (Figure 1). This is nonspecific but could be seen in the setting of thyroiditis. Thyroid laboratory data as seen in Table 2 revealed a hyperthyroid state.

**Table 2:** Thyroid laboratory data TSH: thyroid-stimulating hormone

VARIABLE	Upon Admission	Upon Discharge
TSH (mIU/L)	<0.01 (Low)	<0.01 (Low)
T4 FREE (ng/dl)	2.77 (High)	2.3 (High)
FREE T3 (pg/ml)	9.45 (High)	3.54

The patient had a Burch and Wartofsky score of 50 which is indicative of a thyroid storm (Table 3). Treatment was immediately initiated with propranolol (60 mg) to control adrenergic symptoms such as tachycardia and agitation, propylthiouracil (200 mg) to block hormone synthesis, and hydrocortisone (100 mg) to reduce peripheral T4-T3 conversion and improve vasomotor stability. Lugol’s iodine solution was initiated 1 hour after the antithyroid drugs to stop thyroid hormone synthesis.

**Table 3:** Burch-Wartofsky Point Score (BWPS) HR: heart rate

Thermoregulatory dysfunction	Temperature (°F)	99-99.5	0
		100-100.9	10
		101-101.9	15
		102-102.9	20
		103-103.9	25
		>104	30
Cardiovascular	Tachycardia (HR)	90-109	5
		110-119	10
		120-129	15
		130-139	20
		>140	25
	Atrial Fibrillation	Absent	0
		Present	10
	Congestive Heart Failure	Absent	0
		Mild	5
		Moderate	10
		Severe	15
Gastrointestinal Hepatic Dysfunction	Manifestation	Absent	0
		Moderate (Diarrhea, abdominal pain, nausea, vomiting,)	10
		Severe (Jaundice)	20
Central Nervous System Disturbance	Manifestation	Absent	0
		Mild (Agitation)	10
		Moderate (Delirium, psychosis, lethargy)	20
		Severe (Coma, seizure)	30
Precipitating Event	Status	Absent	0
		Present	10
Interpretation	Thyroid Storm if total score >45		
	Impending Thyroid Storm if score is 25-44		
	Thyroid Storm Unlikely if score is <25		

The patient’s mental status improved the following day. The patient was able to hold a conversion and was alert, oriented, x3. The patient’s free T4 and T3 trended down while thyroid-stimulating hormone (TSH) remained low. The patient was stable upon discharge to a rehabilitation center and was recommended to follow up with an endocrinologist within a week for further management. The patient was discharged with 20 mg of hydrocortisone and 10 mg of methimazole.

## Discussion

Hyperthyroidism is a pathological state characterized by an abnormally increased synthesis of thyroid hormones T3 and T4. This patient had primary hyperthyroidism due to her decreased TSH and increase T3 and T4. Some common causes of primary hyperthyroidism include Graves' disease, toxic adenoma, toxic multinodular goiter and thyroiditis [1]. Thyroid storm usually occurs as a precipitant of bodily stress such as infections, radioiodine therapy, pregnancy, or acute illness such as acute myocardial infarction, stroke, congestive heart failure. This patient had none of the preceding factors that lead to her acute episode of thyroid storm. This patient had undiagnosed hyperthyroidism for at least a year as her family stated that she has symptoms of extreme weight loss, hair loss, dry mouth, fevers, poor oral intake, vertigo and tinnitus. This patient’s acute episode of thyroid storm was a manifestation of untreated thyrotoxicosis. Clinical symptoms of thyroid storm include tachycardia, fever, agitation, change in mental status, and gastrointestinal upset. Elevated thyroid hormone levels can also increase tachyarrhythmias and cardiomyopathies. Patients may present with heart failure or atrial fibrillation. Common medications that can precipitate hyperthyroidism include amiodarone, interferon-alpha and other iodides [2]. The patient was not taking any of the above-listed medications.

In a patient with overt hyperthyroidism, testing for anti-thyrotropin receptor autoantibodies has excellent sensitivity and specificity for Graves’ disease [3]. This result came back positive a week after the patient was discharged. This patient was treated for thyroid storm with propranolol (60 mg) to control adrenergic symptoms, propylthiouracil (200 mg) to block hormone synthesis, hydrocortisone (100 mg) to reduce T4-T3 conversion, and Lugol's iodine. The patient was started on propylthiouracil (PTU) rather than methimazole upon admission because PTU also prevents peripheral conversion of T4 to T3 which would more rapidly reduce circulating T3 levels. The patient was later switched to methimazole after lab value improvement because it has a greater safety profile. In most patients who survive thyroid storm, the clinical improvement is most dramatic and seen within the first 24 hours of treatment [4].

## Conclusions

The patient was treated conventionally for thyroid storm and showed remarkable improvement after one day. Her altered mental status and hallucinations completely went away. Her gastrointestinal symptoms resolved. In summary, because thyroid storm could be the first presentation in a large proportion of patients with hyperthyroidism, delayed treatment could increase mortality. It is important for physicians to be vigilant and have a high index of suspicion to successfully treat thyroid storm before it manifests into multisystem organ failure.
